# Phytochemicals in *Helicobacter pylori* Infections: What Are We Doing Now?

**DOI:** 10.3390/ijms19082361

**Published:** 2018-08-10

**Authors:** Bahare Salehi, Farukh Sharopov, Miquel Martorell, Jovana Rajkovic, Adedayo Oluwaseun Ademiluyi, Mehdi Sharifi-Rad, Patrick Valere Tsouh Fokou, Natália Martins, Marcello Iriti, Javad Sharifi-Rad

**Affiliations:** 1 Medical Ethics and Law Research Center, Shahid Beheshti University of Medical Sciences, Tehran 88777539, Iran; bahar.salehi007@gmail.com; 2 Student Research Committee, Shahid Beheshti University of Medical Sciences, Tehran 22439789, Iran; 3 Department of Pharmaceutical Technology, Avicenna Tajik State Medical University, Rudaki 139, Dushanbe 734003, Tajikistan; shfarukh@mail.ru; 4 Nutrition and Dietetics Department, Faculty of Pharmacy, University of Concepción, Concepción, 4070386 VIII–Bio Bio Region, Chile; mmartorell@udec.cl; 5 Institute of Pharmacology, Clinical Pharmacology and Toxicology, Medical Faculty, University of Belgrade, 11129 Belgrade, Serbia; jolarajkovic@yahoo.com; 6 Functional Foods, Nutraceuticals and Phytomedicine Unit, Department of Biochemistry, Federal University of Technology, Akure 340252, Nigeria; ademiluyidayo@yahoo.co.uk; 7 Department of Medical Parasitology, Zabol University of Medical Sciences, Zabol 61663335, Iran; 8 Department of Biochemistry, Faculty of Science, University of Yaounde 1, Yaounde P.O. Box 812, Cameroon; 9 Faculty of Medicine, University of Porto, Alameda Prof. Hernâni Monteiro, 4200-319 Porto, Portugal; 10 Institute for Research and Innovation in Health (i3S), University of Porto, 4200-135 Porto, Portugal; 11 Department of Agricultural and Environmental Sciences, Milan State University, via G. Celoria 2, 20133 Milan, Italy; 12 Phytochemistry Research Center, Shahid Beheshti University of Medical Sciences, Tehran 11369, Iran; 13 Department of Chemistry, Richardson College for the Environmental Science Complex, The University of Winnipeg, Winnipeg, MB R3B 2G3, Canada

**Keywords:** plant products, *Helicobacter pylori*, opportunistic colonization, phytopharmacology, in vitro/in vivo findings, anti-urease activity

## Abstract

In this critical review, plant sources used as effective antibacterial agents against *Helicobacter pylori* infections are carefully described. The main intrinsic bioactive molecules, responsible for the observed effects are also underlined and their corresponding modes of action specifically highlighted. In addition to traditional uses as herbal remedies, in vitro and in vivo studies focusing on plant extracts and isolated bioactive compounds with anti-*H. pylori* activity are also critically discussed. Lastly, special attention was also given to plant extracts with urease inhibitory effects, with emphasis on involved modes of action.

## 1. Introduction

Plant products, their enriched-derived extracts, and their isolated bioactive molecules have been increasingly studied due their renowned health attributes, largely used in folk medicine over centuries for multiple purposes [1,2,3,4,5,6,7,8,9]. Indeed, phytomedicine is garnering much attention among the medical and scientific communities [10,11,12]. Commercially available synthetic drugs have often been negatively pointed out due to their side effects and related toxicity [13]. In fact, the active molecules used in pharmaceutical formulation are formerly derived from bioactive molecules extracted from plants and other living organisms [14]. Also, a growing number of studies have progressively underlined the multiple bioactive properties conferred by plant formulations [15,16]. Specifically, the antimicrobial effects of multiple plant preparations have been progressively confirmed and supported by both in vitro and in vivo studies and clinical trials [17,18,19,20,21]. Thus, their lower costs, high effectiveness, bioavailability, bioefficacy, and few to no adverse effects have led to intensive research on this topic [22,23,24,25,26,27,28].

Among the various opportunistic infections, those caused by *Helicobacter pylori*, a human opportunistic pathogen, is attracting much attention [29]. In fact, it is widely recognized that this bacterium plays an important role in the etiology of peptic and gastric ulcers and even gastric cancers and gastric lymphomas [29]. About half of the worldwide population is colonized by this bacterium, but there are only about 20% who manifest clinical symptoms, which has been linked to the ability of some *H. pylori* strains to both adapt to host’s immunological responses and to support an ever-changing gastric environment [29]. Relatedly, increasing rates of antibiotic-resistant *H. pylori* strains have been found, and therefore, the search for new eradication strategies and effective antibiotic therapies has become an issue of crucial importance [30]. Hence, research effort is focused on exploring plants as sources of anti-*H. pylori* agents.

Based on these findings, the present report aims to provide an extensive overview of *Helicobacter pylori* infections, namely describing its involvement in triggering gastric cancer and the most common antimicrobials used in *H. pylori* eradication. Special attention is also given to medicinal plants and their corresponding extracts and isolated constituents used as anti-*H. pylori* agents and urease inhibitors. This review was performed by consulting the databases of PubMed, Web of Science, Embase, and Google Scholar (as a search engine); only full-text available articles were considered, and articles published from 2008 to 2018 were prioritized. The search strategy included the combination of following keywords: “*Helicobacter pylori*”, “anti-*Helicobacter*”, “medicinal plant”, “plant extract”, “essential oil”, “bioactive”, “phytochemical”, “antimicrobial”, and “eradication”.

## 2. *Helicobacter pylori* and Gastric Cancer

*H. pylori* infection has been implicated in the development of gastric cancer, a multifactorial disease and a leading cause of mortality. The risk factors for gastric cancer have been shown to include environmental factors and factors that influence host–pathogen interaction, as well as the complex interplay between these factors [31]. Modern lifestyle, high stress levels, smoking and excessive alcohol consumption, nutritional deficiencies, and prolonged use of non-steroidal anti-inflammatory drugs (NSAIDs) are amongst the most relevant etiological environmental factors [32].

This bacterial infection has been linked to the initiation of chronic gastritis that could later lead to adenocarcinoma of the intestine [33]. However, several mechanisms have been proposed to represent the involvement of *H. pylori* infection in tumorigenesis. Several bacterial virulence factors, such as the cytotoxin-associated gene A (CagA) protein, present in the DNA insertion element Cag pathogenicity island (CagPAI), were found to be of prominent importance in carcinogenesis [34]. Likewise, bacterial peptidoglycan can be delivered into gastric epithelial cells, where it activates a phosphoinositide 3-kinase (PI3K)-Akt pathway leading to cell proliferation, migration, and prevention of apoptosis [35]. Furthermore, *H. pylori*-induced gastric inflammation involves the cyclooxygenase-2 (COX2)/prostaglandin E2 (PGE2) pathway and inflammatory marker interleukin 1β (IL-1β), which are important factors triggering chronic active gastritis and adenocarcinoma [31]. Studies have also shown that *H. pylori* infection-induced oxidative stress and DNA damage coupled with dysregulation of E-cadherin/β-catenin/p120 interactions also play critical roles in tumorigenesis [31]. Several environmental and dietary factors have also been suggested to modify *H. pylori*-induced adenocarcinoma [36]. Gastric adenocarcinoma is strongly influenced by dietary salt intake, with high salt intake aggravating tumorigenesis [37].

## 3. Antimicrobials for *H. pylori* Eradication

The success of *H. pylori* eradication markedly depends on the type and duration of treatment, patient compliance to therapy, and antibiotic resistance. For example, because it is difficult to achieve optimal eradication of *H. pylori* infection in patients with peptic ulcers, combinational regimens using two or three antibiotics in addition to a proton pump inhibitor or bismuth are often prescribed to achieve higher eradication rates and to prevent antibiotic resistance emergence [38,39]. These regimens, also known as triple therapies, have cure rates of around 85–90%. They are usually administered for a period of about 10–14 days, in which treatment regimens include the following: (A) bismuth subsalicylate, metronidazole, and tetracycline for 14 days; (B) omeprazole, amoxicillin, and clarithromycin for 10 days; and (C) lansoprazole, amoxicillin, and clarithromycin for either 10 or 14 days.

Unfortunately, the heightening of antimicrobial resistance has been associated with increases in the standard triple therapies failure to eradicate *H. pylori* infection [40]. Hence, research is focusing on developing potent and effective antibacterial regimens that will favor total eradication of the infection. Nonetheless, any eradication treatment comes with some degree of adverse effects, such as nausea, metallic taste, vomiting, skin rash, and diarrhea. Therefore, efforts are being channeled towards the development of effective treatments with few to no side effects.

In the Maastricht V/Florence Consensus Report, 43 experts from 24 countries provided recommendations on the basis of the best available evidence and relevance to the current therapeutic options of management of *H. pylori* infection in the various clinical scenarios [41].

## 4. Plant Extracts and Phytochemicals with Anti-*Helicobacter pylori* Activity

Considering that *H. pylori* infection has been associated with gastrointestinal diseases, including chronic gastritis, peptic ulcer, gastric carcinoma, and mucosa-associated lymphoid tissue lymphoma [42], and that, due to the widespread use of therapeutic agents for the eradication of this bacterium and associated-side effects, increasing rates of *H. pylori* strains with acquired resistance have been discovered. So, the urgent need for alternative has been rekindled and aided by the use of natural drugs [32].

Despite, the newly proposed and used tri-therapy regimens, the cost of acid suppressors and stomach protectors make it inaccessible to the majority of the population [43]. Naturally-derived drugs, including herbs, have been shown to display anti-*H. pylori* activities with minimal side effects, easy accessibility, and affordability [42,44]. In fact, many medicinal plants have been reported in the traditional management of gastrointestinal disorders. Many of these medicinal plants have gone through bioassays to assess their potency against *H. pylori.* Here, the anti-*H. pylori* activity of medicinal plants and isolated bioactive molecules is discussed [45].

Almost all plant parts have been tested for anti-*H. pylori* activity. Plant extract preparations include water (Table 1), essential oils (Table 2), or organic solvents, such as the following: ethanol (Table 3); methanol (Table 4); acetone (Table 5); chloroform (Table 6); petroleum ether (Table 7); methanol/water, ethanol/water, methanol/petroleum, and methanol/dichloromethane extracts (Table 8); and other plant extracts (Table 9).

The susceptibility of *H. pylori* isolates and strains to 543 extracts from 246 plant species was tested by disc diffusion, agar diffusion, agar dilution, and broth microdilution assays. Activity ranged from 1.56–100,000 µg/mL for minimal inhibitory concentration (MIC) and 7–42 mm for inhibition zone diameters (IZDs). However, disparities were observed among the methods used and the tested concentrations: some extracts were tested at very high concentrations (100,000 µg/mL) that might have resulted in biased conclusions. Though many plants (246 species) showed anti-*H. pylori* activity in vitro, very few have been screened for activity in animal models.

Organic extracts of *Carum carvi*, *Xanthium brasilicum*, and *Trachyspermum copticum* have demonstrated antibacterial activity against 10 clinical isolates of *H. pylori* [46]. In addition, ethanolic extracts of *Cuminum cyminum* and propolis exhibited significant in vitro inhibitory effect against *H. pylori* and, therefore, could be considered a valuable support in the treatment of infection, even contributing to the development of new and safer agents for inclusion in anti-*H. pylori* therapy regimens [47]. Some popular plant species used in Brazilian cuisine and folk medicine in the treatment of gastrointestinal disorders were also investigated for their antibacterial effects, among which *Bixa orellana*, *Chamomilla recutita*, *Ilex paraguariensis*, and *Malva sylvestris* were the most effective against *H. pylori* [48].

Bioactive plant compounds were also tested for their anti-*H. pylori* potency (Table 10), namely those isolated from the *Allium sativum* (cloves), *Convolvulus austro-aegyptiacu* (aerial parts), *Glycyrrhiza glabra* (roots), *Hydrastis canadensis* (rhizomes), *Sanguinaria canadensis* (rhizomes), and *Tinospora sagittata* (aerial parts) species. Berberine, a benzylisoquinoline alkaloid, isolated from *Hydrastis canadensis*, revealed the lowest MIC value (0.78 μg/mL), being therefore considered the most effective bioactive compound, followed by diallyl tetrasulfide (3–6 μg/mL), allicin (4 μg/mL), and palmatine (3.12–6.25 μg/mL) isolated from *Allium sativum* and *Tinospora sagittate*, respectively.

## 5. In Vivo Findings

*H. pylori* colonization is increasingly being associated with a heightened risk of developing upper gastrointestinal tract diseases. Despite many plant extracts having demonstrated a prominent *H. pylori* inhibition capacity in culture, it is of crucial importance to assess their in vivo efficacy, because it is pivotal to ascertain their effective antibacterial potency. However, a relatively low number of medicinal plants have been investigated to date for in vivo activity, as discussed below.

*Paeonia lactiflora* root extract (100 µg/mL) showed a complete inhibition of *H. pylori* colonization (4–5 × 10^5^ colony forming unit (CFU)), being the antibacterial potential equivalent to of ampicillin used as positive control (10 µg/mL) (2–4 × 10^5^ CFU) [98]. Time course viability experiments were also performed in simulated gastric environments to assess the anti-*H. pylori* activity of garlic (*Allium sativum*) oil (16 and 32 µg/mL). A rapid anti-*H. pylori* action in artificial gastric juice was found. Nevertheless, the anti-*H. pylori* activity displayed by garlic oil was noticeably affected by food materials and mucin, despite the fact that a substantial activity remained under simulated gastric conditions [65]. Also, *H. pylori*-inoculated Swiss mice receiving 125, 250, or 500 mg/kg of *Bryophyllum pinnutum* or ciprofloxacin (500 mg/kg) for 7 days, showed a significant reduction of *H. pylori* colonization on gastric tissue from 100% to 17%. In addition, the highest *B. pinnatum* extract tested (85.91 ± 52.91 CFU) and standard drug ciprofloxacin (25.74 ± 16.15 CFU) also reduced significantly (*p* < 0.05) the bacterial load of gastric mucosa as compared with untreated infected mice (11883 ± 1831 CFU) [74]. On the other hand, *Eryngium foetidum* methanol extract (381.9 ± 239.5 CFU) and positive control ciprofloxacin (248 ± 153.2 CFU) significantly reduced the bacterial load in gastric mucosa at the same dose (500 mg/kg) compared with untreated and inoculated mice (14350 ± 690 CFU) [73].

*Hippocratea celastroides* hydroethanolic root-bark extract, a widely used plant against gastric and intestinal infections, also showed anti-*H. pylori* efficacy in naturally infected dogs. In a study of 18 experimental dogs treated with a dose of 93.5–500 mg/kg of *H. celastroides* extract in weight and 19 infected dogs receiving amoxicillin–clarithromycin–omeprazole (control treatment), the results showed effectiveness of 33.3 and 55% in the experimental and control groups, respectively [99].

On the other hand, Ye et al. [95], aiming to investigate the in vivo bactericidal effects of *Chenopodium ambrosioides* L. against *H. pylori*, randomly assigned *H. pylori*-infected mice into plant extract group, triple therapy control (lansoprazole, metronidazole, and clarithromycin), blank control, and *H. pylori* control groups. The obtained eradication ratios, determined by rapid urease tests (RUTs) and histopathology, were, respectively, 60% (6/10) using RUT and 50% (5/10) using histopathology for the test group and both 70% (7/10) for the control group. In addition, the histopathologic evaluation revealed a massive bacterial colonization on the gastric mucosa surface and slight mononuclear cells infiltration after *H. pylori* inoculation, but no obvious inflammation or other pathologic changes in gastric mucosa were stated between the *C. ambrosioides*-treated mice and the standard therapy.

*Tinospora sagittata* and its main component, palmatine, showed in vitro bactericidal effects on *H pylori* strains, with both MIC and minimal bactericidal concentration (MBC) values of 6250 μg/mL, whereas palmatine’s MIC value against *H. pylori* SCYA201401 was 6.25 μg/mL and against *H. pylori* SS1 was 3.12 μg/mL. The time-kill kinetic study evidenced a dose-dependent and progressive decline in the numbers of viable bacteria up to 40 h. *H. pylori*-infected mice treated with extract, palmatine, or control therapy (omeprazole, clarithromycin, and amoxicillin), presented eradication ratios of, respectively, 80%, 50%, and 70%. The anti-*H. pylori* activity found in *T. sagittata* extracts and its major constituent, palmatine, both in culture and animal models, clearly highlights the antibacterial potential of this plant in the treatment of both infected humans and animals [42].

Total alkaloids fraction activity (TASA) of *Sophora alopecuroides* L., widely used in herbal remedies against stomach-associated diseases, were also investigated on 120 *H. pylori*-infected BALB/c mice mouse gastritis. A total of 100 infected mice were randomly assigned into 10 treatment groups: group I (normal saline); group II (bismuth pectin); group III (omeprazole); group IV (TASA 2 mg/day); group V (TASA 4 mg/day); group VI (TASA 5 mg/day); group VII (TASA + bismuth pectin); group VIII (TASA + omeprazole); group IX (bismuth pectin + clarithromycin + metronidazole); and group X (omeprazole + clarithromycin + metronidazole). The mice were sacrificed 4 weeks after treatment. Real-time PCR was used to detect 16sDNA of *H. pylori* to test both the colonization and mice clearance of bacteria of each treatment. Hematoxylin and eosin staining and immunostaining of mice gastric mucosa were also used to observe the general inflammation and related factors: IL-8, COX2, and nuclear factor-kappa B (NF-κB) expression changed after treatments. TASA combined with omeprazole or bismuth pectin showed promising antimicrobial activity against *H. pylori*, as well as conventional triple therapy. Indeed, hematoxylin and eosin staining and immune-staining of mice gastric mucosa evidenced that the inflammation on mice gastric mucosal membrane were also clearly relieved in TASA combined treatments and conventional triple therapy compared with normal saline-treated mice. Accordingly, from immunohistochemistry results, *H. pylori*-induced IL-8, COX2, and NF-κB were consistently suppressed in the seventh, eighth, ninth, and tenth groups to a certain extent [100].

Pastene et al. [101] investigated the inhibitory effects of a standardized apple peel polyphenol-rich extract (*Malus pumila* Mill., cited as *Malus domestica*) against *H. pylori* infection and vacuolating bacterial toxin (VacA)-induced vacuolation and found that the preparation significantly prevented vacuolation in HeLa cells with an IC_50_ value of 390 μg gallic acid equivalents (GAE)/mL and an in vitro anti-adhesive effect against *H. pylori*. A significant inhibition was also stated with 20–60% reduction of *H. pylori* attachment at concentrations between 0.250 and 5 mg GAE/mL. In a short-term infection model (C57BL6/J mice), doses of 150 and 300 mg/kg/day showed an inhibitory effect on *H. pylori* attachment. Orally administered apple peel polyphenols also showed an anti-inflammatory effect on *H. pylori*-associated gastritis, lowering malondialdehyde levels and gastritis scores.

Kim et al. [102] investigated the GutGard™ ability (a flavonoid rich, *Glycyrrhiza glabra* root extract) to inhibit *H. pylori* growth both in Mongolian gerbils and C57BL/6 mouse models. Infected male Mongolian gerbils were orally treated once daily 6 times/week for 8 weeks with 15, 30, and 60 mg/kg GutGard™. Bacterial identification in the biopsy samples of gastric mucosa, via urease, catalase, and ELISA, as well as immunohistochemistry revealed a dose-dependent inhibition of *H. pylori* colonization in gastric mucosa by GutGard™. As well, the administration of 25 mg/kg GutGard™ in *H. pylori*-infected C57BL/6 mice significantly reduced *H. pylori* colonization in gastric mucosa, suggesting its usefulness in *H. pylori* infection prevention.

*Calophyllum brasiliense* stem bark preparations are popular remedies for the treatment of chronic ulcers. A current report evidenced gastroprotective, gastric acid inhibitory properties and anti-*H. pylori* activity in culture (MIC = 31 µg/mL) [75]. Hydroethanolic (50, 100, and 200 mg/kg) and dichloromethane (100 and 200 mg/kg) fractions-treated Wistar rats ulcerated by acetic acid and inoculated with *H. pylori*, showed a marked delay in ulcer healing and reduced the ulcerated area in a dose-dependent manner [75]. While the dichloromethane fraction, at 200 mg/kg, increased PGE2 levels, both the hydroethanolic and dichloromethane fractions decreased the number of urease-positive animals, as confirmed by the reduction of the *H. pylori* presence in histopathological analysis. This aspect suggests that the antiulcer activity of *C. brasiliense* is partly linked with its anti-*H. pylori* efficacy [75]. Also, phenolic-rich oregano (*Origanum vulgare*) and cranberry (*Vaccinium macrocarpon*) extracts showed a prominent ability to inhibit *H. pylori* through urease inhibition and disruption of energy production by inhibition of proline dehydrogenase at the plasma membrane [103].

## 6. Urease Inhibition

The current therapies are challenged by the considerable number of emerging *H. pylori*-resistant strains. This fact has driven the need for alternative anti-*H. pylori* therapies that ideally should have good stability and low toxicity and to be able to inhibit urease activity [62]. It has been shown that *H. pylori* urease activity is crucial in bacterial survival and pathogenesis [104].

The inhibitory potency of some anti-*H. pylori* medicinal plants has been reported [62] and even investigated by some authors in the involved mechanisms of antibacterial action of those plant products [63].

Table 11 briefly shows the studied plant extracts with prominent anti-urease activity. Amin et al. [49] demonstrated that the methanolic and acetone extracts of some medicinal plants were able to inhibit urease activity. In fact, *Acacia nilotica* flower methanol and acetone extracts evidenced anti-*H. pylori* activity, being MIC values of 8–64 μg/mL and 4–64 μg/mL, respectively. Both extracts inhibited urease activity at 8.2–88.2% and 9.2–86.6%. *Calotropis procera* leaf and flower methanol and acetone extracts, with MIC values of 16–256 μg/mL, 32–256 μg/mL, and 8–128 μg/mL also displayed urease inhibitory effects, being, respectively, 12.2–48.2% and 7.2–58.2% for leaf and 9.3–68.2% for flower acetone extracts [49]. While *A. nilotica* extract exerted a competitive inhibition, that of *C. procera* extract displayed a mixed type of inhibition [49]. In addition, *Casuarina equisetifolia* fruit methanol extract, with MIC values varying from 128–512 μg/mL, also displayed 12.2–86.2% inhibition of urease activity [49].

In another study, *Camellia sinensis* young non-fermented and semi-fermented shoot extracts, presented inhibition zone diameter (IZD) and MBC of, respectively, 22.5 mm at 20–60 μg/disk and 4 mg/mL, and 18 mm at 20–60 μg/disk and 5.5 mg/mL. They both inhibited Ure A and Ure B subunits production at 2.5 and 3.5 mg/mL [94]. Also, the *Chamomilla recutita* flower extract, which inhibited *H. pylori* growth at an MIC_90_ value of 125 mg/mL and a MIC_50_ value of 62.5 mg/mL, was able to inhibit the urease production [105]. In the same line, the methanol fraction of *Euphorbia umbellata* bark extract inhibited both *H. pylori* growth (44.6% inhibition) at 256 μg/mL and urease activity (78.6% inhibition) at 1024 μg/mL [77]. Moreover, the *Peumus boldus* flower aqueous extract showed anti-adherent activity against *H. pylori* and inhibited urease activity with an IC_50_ value of 23.4 μg GAE/mL [61]. The aqueous extract of *Teminalia chebula* fruit showed activity with MIC and MBC values of 125 mg/mL and 150 mg/mL, respectively, and inhibited *H. pylori* urease activity at a concentration of 1–2.5 mg/mL [63].

## 7. Conclusions and Future Perspectives

Overall, the report suggests that the studied plant extracts possess anti-*H. pylori* activity, strengthening the claims made by traditional medicine practitioners about their putative anti-ulcerative properties. However, very few of them were investigated for efficacy in animal models or the ability to inhibit urease activity. Further studies are warranted for efficacy studies in animal models, elucidation of effective modes of action (including urease inhibition), and clinical trials in human being.

## Figures and Tables

**Table 1:** Plant aqueous extracts with anti-*Helicobacter pylori* activity.

Species	Family	Parts	Anti-*H. pylori* Potency	Ref.
*Acacia nilotica* (L.) Delile	*Leguminosae*	Flowers	MIC = 8–64 μg/mL	[49]
*Adhatoda vasica* Nees	*Acanthaceae*	Whole plant	MIC = 16–512 μg/mL	[49]
*Alepidea amatymbica* Eckl. and Zeyh	*Apiaceae*	Roots/Rhizomes	IZD = 8.0 ± 8.2 mm	[50,51]
*Amphipterygium adstringens* (Schltdl.) Standl.	*Anacardiaceae*	Aerial parts	MIC = 62.5–125 µg/mL	[52]
*Annona cherimola* Mill.	*Annonaceae*	Leaves/Stem	MIC = 500 µg/mL	[52]
*Artemisia ludoviciana* Nutt. subsp. *mexicana* (Willd. ex Spreng.) Fernald	*Compositae*	Leaves/stems	MIC = 125 µg/mL	[52]
*Bridelia micrantha* (Hochst.) Baill.	*Phyllanthaceae*	Bark	IZD = 0–15 mm; MIC_50_ = 48–313 mg/mL; MIC_90_ = 78 ≥ 625 µg/mL	[53]
*Buddleja perfoliata* Kunth	*Scrophulariaceae*	Aerial parts	MIC = 500 µg/mL	[52]
*Calandrinia ciliata* (Ruiz and Pav.) DC. (cited as *Calandrinia micrantha* Schltdl.)	*Portulacaceae*	Leaves/Stems	MIC = 1000 µg/mL	[52]
*Calotropis procera* (Aiton) W.T. *Aiton*	*Apocynaceae*	Leaves	MIC = 16–256 μg/mL	[49]
		Flowers	MIC = 8–256 μg/mL	[49]
*Campyloneurum amphostenon* (Kunze ex Klotzsch) Fée	*Polypodiaceae*	Aerial parts	MIC = 1000 µg/mL	[52]
*Casuarina equisetifolia* L.	*Casuarinaceae*	Fruit	MIC = 128–1024 μg/mL	[49]
*Chenopodium incisum* Poir. (cited as *Teloxys graveolens* (Willd.) W. A. Weber)	*Amaranthaceae*	Aerial parts	MIC = 250 µg/mL	[52]
*Cichorium intybus* L.	*Asteraceae*	Root	IZD < 9 mm	[47]
*Cinnamomum zeylanicum* Blume	*Lauraceae*	Bark	IZD < 9 mm	[47]
*Cistus laurifolius* L.	*Cistaceae*	Flowers	MIC = 62.5–125 µg/mL	[54]
*Citrus reticulata* Blanco	*Rutaceae*	Fruit shell	MIC = 100 µg/mL	[55]
*Cocculus hirsutus* (L.) Diels.	*Menispermaceae*	Leaves	IZD = 22 mm (200–1000 μg/mL)	[56]
*Combretum molle* R. Br. Ex G. Don	*Combretaceae*	Bark	IZD = 2.7 ± 5.5 mm	[50,51]
*Coriandrum sativum* L.	*Apiaceae*	Seed	IZD = 9 mm; MIC = 1.25–5 mg/mL	[47]
*Corydalis yanhusuo W.T. Wang*	*Papaveraceae*	Stem	MIC = 100 µg/mL	[55]
*Cuminum cyminum* L.	*Apiaceae*	Seed	IZD < 9 mm	[47]
*Cuphea aequipetala* Cav.	*Lythraceae*	Aerial parts	MIC = 125 μg/mL	[57]
			MIC = 125 µg/mL	[52]
*Cynara scolymus* L.	*Asteraceae*	Leaves	IZD = 18 mm; MIC= 1.25–5 mg/mL	[47]
*Cyrtocarpa procera* Kunth	*Anacardiaceae*	Bark	MIC = 125 µg/mL	[58]
			MIC = 250 µg/mL	[52]
*Desmos cochinchinensis* Lour.	*Annonaceae*	Leaves	IZD = 10.0 ± 0.6 mm (240 µg/disc)	[59]
*Dysphania ambrosioides* (L.) Mosyakin and Clemants (cited as *Teloxys ambrosioides* (L.) W. A. Weber)	*Amaranthaceae*	Aerial parts	MIC = 1000 µg/mL	[52]
*Elettaria cardamomum* (L.) Maton.	*Zingiberaceae*	Seeds	IZD < 9 mm	[47]
*Eryngium carlinae* F. Delaroche	*Apiaceae*	Aerial parts	MIC = 1000 µg/mL	[52]
*Eugenia caryophyllata* Thunb	*Myrtaceae*	Flower	MIC = 60 µg/mL	[55]
*Eupatorium petiolare* Moc. ex DC.	*Compositae*	Aerial parts	MIC = 500 µg/mL	[52]
*Fagoniaar abica* L.	*Zygophyllaceae*	Whole plant	MIC = 16–256 μg/mL	[49]
*Foeniculum vulgare* Mill. var. dulce DC	*Apiaceae*	Seed	IZD < 9 mm; MIC = 5–10 mg/mL	[47]
*Fritillaria thunbergii* Miq.	*Liliaceae*	Stem	MIC = 40 µg/mL	[55]
*Garcinia kola* Heckel	*Guttiferae*	Seeds	IZD = 1.0 ± 2.6 mm	[50,51]
*Geum iranicum* Khatamsaz	*Rosaceae*	Root	IZD = 24–35 mm (100 µg/mL)	[60]
*Gnaphalium canescens DC.*	*Compositae*	Aerial parts	MIC = 500 µg/mL	[52]
*Grindelia inuloides* Willd.	*Compositae*	Aerial parts	MIC = 500 µg/mL	[52]
*Hesperozygis marifolia* Epling	*Lamiaceae*	Aerial parts	MIC = 1000 µg/mL	[52]
*Heterotheca inuloides* Cass.	*Compositae*	Aerial parts	MIC = 500 µg/mL	[52]
*Juniperus communis* L.	*Cupressaceae*	Berry	IZD < 9 mm	[47]
*Larrea tridentata* (Sessé and Moc. ex DC.) Coville	*Zygophyllaceae*	Aerial parts	MIC = 500 µg/mL	[52]
*Ligusticum striatum* DC (cited as *Ligusticum chuanxiong* Hort.)	*Apiaceae*	Root	MIC = 100 µg/mL	[55]
*Lippia graveolens* Kunth (cited as *Lippia berlandieri* Schauer)	*Verbenaceae*	Aerial parts	MIC = 1000 µg/mL	[52]
*Ludwigia repens* J. R. Forst.	*Onagraceae*	Aerial parts	MIC = 125 µg/mL	[52]
*Machaeranthera riparia* (Kunth) A.G. Jones	*Compositae*	Aerial parts	MIC = 1000 µg/mL	[52]
*Machaeranthera tanacetifolia* (Kunth) Nees	*Compositae*	Aerial parts	MIC = 1000 µg/mL	[52]
*Mentha × piperita* L.	*Lamiaceae*	Leaves	IZD < 9 mm	[47]
*Mirabilis jalapa* L.	*Nyctaginaceae*	Aerial parts	MIC = 250 µg/mL	[52]
*Monarda citriodora* var. *austromontana* (Epling) B. L. *Turner* (cited as *Monarda austromontana* Epling)	*Lamiaceae*	Aerial parts	MIC = 500 µg/mL	[52]
*Olea europaea* L.	*Oleaceae*	Leaves/Stem	MIC = 125 µg/mL	[52]
*Origanum vulgare* L.	*Lamiaceae*	Leaves	IZD = 25 mm; MIC = 0.6–2.5 mg/mL	[47]
*Orthosiphon aristatus* (Blume) Miq. (cited as *Orthosiphon stamineus* Benth)	*Lamiaceae*	Leaves	IZD = 9.0 ± 1.3 mm (240 µg/disc)	[59]
		Stem	IZD = 8.0 ± 0.1 mm (240 µg/disc)	[59]
*Peumus boldus* Mol.	*Monimiaceae*	Leaves	>1500 μg/mL	[61]
*Plantago major* L.	*Plantaginaceae*	Aerial parts	MIC = 1000 µg/mL	[52]
*Priva grandiflora* (Ortega) Moldenke	*Verbenaceae*	Aerial parts	MIC = 250 µg/mL	[52]
*Prunus avium* L.	*Rosaceae*	Peduncles	IZD = 9 mm; MIC = 5–10 mg/mL	[47]
*Rosmarinus officinalis* L.	*Lamiaceae*	Leaves	IZD < 9 mm	[47]
*Ruta chalepensis* L.	*Rutaceae*	Leaves	MIC = 1000 µg/mL	[52]
*Salvia officinalis* L.	*Lamiaceae*	Leaves	IZD = 10 mm; MIC = 1.25–10 mg/mL	[47]
*Sclerocarya birrea* A. Rich Hochst	*Anacardiaceae*	Stem bark	MIC = 0.16–2.5 mg/mL; IZD = 15.0 ± 2.7 mm	[50,51]
*Tagetes lucida* Cav.	*Compositae*	Aerial parts	MIC = 500 µg/mL	[52]
*Tecoma stans* (L.) Juss. ex Kunth	*Bignoniaceae*	Aerial parts	MIC = 1000 µg/mL	[52]
*Terminalia catappa* L.	*Combretaceae*	Aerial parts	MIC = 125 µg/mL	[62]
*Terminalia chebula* Retz	*Combretaceae*	Fruit	MIC = 125 mg/mL; MBC = 150 mg/mL	[63]
*Thymus serpyllum* L.	*Lamiaceae*	Aerial parts	IZD = 10 mm; MIC = 1.25–10 mg/mL	[47]
*Tillandsia usneoides* L.	*Bromeliaceae*	Aerial parts	MIC = 1000 µg/mL	[52]
*Tinospora sagittata* Gagnep.	*Menispermaceae*	Root	MIC = 100 µg/mL	[55]
*Tithonia diversifolia* (Hemsl.) A.G.	*Compositae*	Aerial parts	MIC = 500 µg/mL	[52]
*Verbena carolina* L.	*Verbenaceae*	Aerial parts	MIC = 62.5–125 µg/mL	[52]
*Zingiber officinale* Roscoe	*Zingiberaceae*	Rhizome	IZD = 9 mm; MIC = 2.5–5 mg/mL	[47]

MIC, minimal inhibitory concentration; IZD, inhibition zone diameter; MBC, minimal bactericidal concentration.

**Table 2:** Plant essential oils with anti-*H. pylori* activity.

Species	Family	Parts	Anti-*H. pylori* Potency	Ref.
*Abies mariesii Mast.* (cited as *Abies maritima*)	*Pinaceae*	Pine	IZD = 22 ± 2 mm (500 µg/disc)	[64]
			IZD = 14 ± 1 mm (500 µg/disc)	[64]
*Allium sativum* L.	*Amaryllidaceae*	Cloves	8–32 μg/mL	[65]
*Artemisia dracunculus L.*	*Compositae*	Tarragon	IZD = 7 ± 0 mm (500 µg/disc)	[64]
*Carum carvi* L.	*Apiaceae*	Caraway	IZD = 12 ± 0 mm (500 µg/disc)	[64]
*Cinnamomum zeylanicum* Blume	*Lauraceae*	Bark	MIC = 0.3 μL/mL; IZD = 24.8 mm	[66]
			IZD = 63 ± 0.5 mm (500 µg/disc)	[64]
			IZD = 45 ± 5 mm (500 µg/disc)	[64]
*Cistus ladanifer* L.	*Cistaceae*	Cistus	IZD = 8 ± 0 mm (500 µg/disc)	[64]
			IZD = 16 ± 1.5 mm (500 µg/disc)	[64]
*Citrus aurantium* L.	*Rutaceae*	Orange blossom	>88% inhibition (0.3 μL/mL)	[66]
			IZD = 12 ± 0 mm (500 µg/disc)	[64]
			IZD = 16 ± 0 mm (500 µg/disc)	[64]
*Citrus limon* (L.) Burm. f.	*Rutaceae*	Lemon	IZD = 16 ± 0 mm (500 µg/disc)	[64]
			IZD = 14 ± 10 mm (500 µg/disc)	[64]
*Citrus paradise* Macfad	*Rutaceae*	White grapefruit	IZD = 29 ± 2.5 mm (500 µg/disc)	[64]
			IZD = 17 mm (500 µg/disc)	[64]
		Grapefruit	IZD = 13 ± 0.5 mm (500 µg/disc)	[64]
		Tea tree	IZD = 9 ± 0 mm (500 µg/disc)	[64]
*Cupressus sempervirens* L.	*Cupressaceae*	Cypress	IZD = 19 ± 3.5 mm (500 µg/disc)	[64]
			IZD = 11 ± 10 mm (500 µg/disc)	[64]
*Cymbopogon citratus* (DC.) Stapf	*Poaceae*	Lemongrass	IZD = 32 ± 7 mm (500 µg/disc)	[64]
			IZD = 23 ± 0.05 mm (500 µg/disc)	[64]
*Daucus carota* L.	*Apiaceae*	Carrot seed	IZD = 8 ± 0.5 mm (500 µg/disc)	[64]
			IZD = 16 ± 1.5 mm (500 µg/disc)	[64]
*Dittrichia viscosa* (L.) Greuter subsp. *revoluta*	*Asteraceae*	Aerial parts	0.33 μL/mL *	[67]
*Eucalyptus globulus* L.	*Myrtaceae*	Eucalyptus	IZD = 10 ± 1 mm (500 µg/disc)	[64]
			IZD = 12 ± 10 mm (500 µg/disc)	[64]
*Eugenia caryophyllus* (*Spreng.*) Bullock and S. G. Harrison	*Myrtaceae*	Clove-bud	IZD = 13 ± 2.5 mm (500 µg/disc)	[64]
		Clove-leaf	IZD = 25 ± 5 mm (500 µg/disc)	[64]
*Heracleum persicum* L.	*Apiaceae*	Fruits	>88% inhibition (0.3 μL/mL)	[66]
*Juniperus communis* L.	*Cupressaceae*	Berry	IZD = 14 ± 0.5 mm (500 µg/disc)	[64]
			IZD = 10 ± 1 mm (500 µg/disc)	[64]
*Leptospermum scoparium* J. R. Forst and G. Forst	*Myrtaceae*	Manuka	IZD = 23 ± 3 mm (500 µg/disc)	[64]
*Aloysia citriodora* Palau (cited as *Lippia citriodora*)	*Verbenaceae*	Aerial parts	IZD = 29 ± 2 mm (500 µg/disc)	[64]
*Matricaria chamomilla* L. (cited as *Matricaria recutita*)	*Compositae*	Flowers	IZD = 7 ± 0 mm (500 µg/disc)	[64]
			IZD = 15 ± 10 mm (500 µg/disc)	[64]
*Melaleuca alternifolia* Cheel.	*Myrtaceae*	Tea tree	IZD = 9 ± 0.3 mm (500 µg/disc)	[64]
*Ocimum basilicum* L.	*Lamiaceae*	Aerial parts	IZD = 9 ± 0.3 mm (500 µg/disc)	[64]
			IZD = 8 ± 0.5 mm (500 µg/disc)	[64]
*Origanum vulgare* L.	*Lamiaceae*	Leaves	IZD = 19 ± 4 mm (500 µg/disc)	[64]
*Pimpinella anisum* L.	*Apiaceae*	Anise	IZD = 12 ± 10 mm (500 µg/disc)	[64]
*Salvia sclarea* L.	*Lamiaceae*	Aerial parts	IZD = 10 ± 2 mm (500 µg/disc)	[64]
			IZD = 10 ± 10 mm (500 µg/disc)	[64]
*Salvia officinalis* L.	*Lamiaceae*	Leaves	IZD = 7 ± 0 mm (500 µg/disc)	[64]
*Sassafras officinale* Siebold	*Lauraceae*	Aerial parts	IZD = 7 ± 0 mm (500 µg/disc)	[64]
*Satureja montana* L.	*Lamiaceae*	Savory	IZD = 25 ± 5 mm (500 µg/disc)	[64]
			IZD = 13 ± 5 mm (500 µg/disc)	[64]
*Syzygium aromaticum* (L.) Merr. and L. M. Perry	*Myrtaceae*	Buds	>88% inhibition (0.3 μL/mL)	[66]
*Thymus vulgaris* L.	*Lamiaceae*	Thyme	IZD = 15 ± 5 mm (500 µg/disc)	[64]
			IZD = 12 ± 10 mm (500 µg/disc)	[64]
*Thymus zygis* L.	*Lamiaceae*	Red thyme	IZD = 19 ± 0.5 mm (500 µg/disc)	[64]
*Zataria multiflora* Boiss.	*Lamiaceae*	Aerial parts	MIC = 0.3 μL/mL IZD = 23.6 mm	[66]

* Initial population of 8.52 ± 0.30 log10 colony forming unit (CFU)/mL reduced to 7.67 ± 0.22 log10CFU/mL; MIC, minimal inhibitory concentration; IZD, inhibition zone diameter.

**Table 3:** Plant ethanolic extracts with anti-*H. pylori* activity.

Species	Family	Parts	Anti-*H. pylori* Potency	Ref.
*Abrus cantoniensis* Bge.	*Leguminosae*	Aerial parts	MIC = 40 µg/mL	[55]
*Alepidea Amatymbica* Eckl. and Zeyh	*Apiaceae*	Roots/rhizomes	IZD = 6.7 ± 6.7 mm	[50,51]
*Amomum villosum* Lour.	*Zingiberaceae*	Fruit	MIC = 100 µg/mL	[55]
*Bixa orellana* L.	*Bixaceae*	Seed	MIC ≤ 625–1250 μg/mL	[48]
*Bupleurum chinense* DC.	*Apiaceae*	Aerial parts	MIC = 60 µg/mL	[55]
*Chamomilla recutita* (L.) Rauschert	*Compositae*	Inflorescences	MIC ≤ 625 μg/mL	[48]
*Cichorium intybus* L.	*Asteraceae*	Root	IZD = 12 mm; MIC = 1.25–10 mg/mL	[47]
*Cinnamomum zeylanicum* Blume	*Lauraceae*	Bark	IZD = 20 mm; MIC = 1.25–5 mg/mL	[47]
*Citrus reticulata* Blanco	*Rutaceae*	Fruit shell	MIC = 60 µg/mL	[55]
*Combretum molle* R. Br. Ex G. Don	*Combretaceae*	Bark	IZD = 12.9 ± 4.7 mm	[50,51]
*Convolvulus austro-aegyptiacu* Abdallah and Saad	*Convolvulaceae*	Aerial parts	MIC = 100–200 µg/mL	[67]
*Coriandrum sativum* L.	*Apiaceae*	Seed	IZD = 12 mm; MIC = 5–10 mg/mL	[47]
*Corydalis yanhusuo* W.T. Wang	*Papaveraceae*	Stem	MIC = 60 µg/mL	[55]
*Cuminum cyminum* L.	*Apiaceae*	Seed	IZD = 14 mm; MIC= 0.075–0.6 mg/mL	[47]
*Cynara scolymus* L.	*Asteraceae*	Leaves	IZD = 25 mm; MIC = 0.15–0.6 mg/mL	[47]
*Elettaria cardamomum* (L.) Maton.	*Zingiberaceae*	Seed	IZD = 18 mm; MIC = 0.6–2.5 mg/mL	[47]
*Eugenia caryophyllata* Thunb	*Myrtaceae*	Flower	MIC = 40 µg/mL	[55]
*Foeniculum vulgare* Mill. var. *dulce* DC	*Apiaceae*	Seed	IZD < 9 mm	[47]
*Fritillaria thunbergii* Miq.	*Liliaceae*	Stem	MIC = 40 µg/mL	[55]
*Garcinia kola* Heckel	*Guttiferae*	Seeds	MIC = 0.63–5 mg/mL; IZD = 9.2 ± 7.2 mm	[50,51]
*Hippophae rhamnoides* L.	*Elaeagnaceae*	Leaves	MIC = 60 µg/mL	[55]
*Ilex paraguariensis* A. St.-Hil.	*Aquifoliaceae*	Green leaves	MIC ≤ 625–5000 μg/mL	[48]
		Roasted leaves	MIC ≤ 625–5000 μg/mL	[48]
*Juniperus communis* L.	*Cupressaceae*	Berry	IZD = 10 mm; MIC = 1.25–10 mg/mL	[47]
*Ligusticum striatum* DC (cited as *Ligusticum chuanxiong*)	*Apiaceae*	Root	MIC = 60 µg/mL	[55]
*Lysimachia christinae* Hance	*Primulaceae*	Whole plant	MIC = 100 µg/mL	[55]
*Magnolia officinalis* Rehd. et Wils.	*Magnoliaceae*	Bark	MIC = 60 µg/mL	[55]
*Malva sylvestris* L.	*Malvaceae*	Leaves and inflorescences	MIC ≤ 625–5000 μg/mL	[48]
*Melia azedarach* L. (cited as *Melia toosendan*)	*Meliaceae*	Fruit	MIC = 100 µg/mL	[55]
*Mentha × piperita* L.	*Lamiaceae*	Leaves	IZD < 9 mm	[47]
*Piper longum* L.	*Piperaceae*	Spike	MIC = 100 µg/mL	[55]
*Prunus avium* L.	*Rosaceae*	Peduncles	IZD = 10 mm; MIC = 1.25–10 mg/mL	[47]
*Rosmarinus officinalis* L.	*Lamiaceae*	Leaves	IZD = 20 mm; MIC = 1.25–10 mg/mL	[47]
*Salvia officinalis* L.	*Lamiaceae*	Leaves	IZD = 14 mm; MIC= 1.25–5 mg/mL	[47]
*Saussurea costus* (Falc.) Lipsch. (cited as *Saussurea lappa*)	*Compositae*	Root	MIC = 40 µg/mL	[55]
*Schisandra chinensis* Baill.	*Schisandraceae*	Fruit	MIC = 60 µg/mL	[55]
*Sclerocarya birrea* A. Rich Hochst	*Anacardiaceae*	Stem bark	IZD = 3.3 ± 5.0 mm	[50,51]
*Thymus serpyllum* L.	*Lamiaceae*	Aerial parts	IZD = 22 mm; MIC = 1.25–10 mg/mL	[47]
*Tinospora sagittata* Gagnep.	*Menispermaceae*	Aerial parts	MIC/MBC = 6250 μg/mL	[42]
*Trigonella foenum-graecum* L.	*Leguminosae*	Seed	MIC = 40 µg/mL	[55]
*Zingiber officinale* Roscoe	*Zingiberaceae*	Rhizome	IZD = 25 mm; MIC = 0.075–0.6 mg/mL	[47]

MIC, minimal inhibitory concentration; IZD, inhibition zone diameter.

**Table 4:** Plant methanolic extracts with anti-*H. pylori* activity.

Species	Family	Parts	Anti-*H. pylori* Potency	Ref.
*Acacia nilotica* (L.) Delile	*Leguminosae*	Leaves	MIC = 8–128 μg/mL	[49]
		Flowers	MIC = 8–64 μg/mL	[49]
*Acanthus montanus* (Nees) T. Anders	*Acanthaceae*	Leaves stalk	IZD = 6–22 mm (25µg/disc)	[68]
*Achillea millefolium* L.	*Compositae*	Aerial parts	MIC = 1.56–100 µg/mL	[69]
*Adhatoda vasica* Nees	*Acanthaceae*	Whole plant	MIC = 64–512 μg/mL	[49]
*Aframomum pruinosum* Gagnepain	*Zingiberaceae*	Seed	MIC = 128 μg/mL	[70]
*Ageratum conyzoides* L.	*Compositae*	Whole plant	IZD = 6–22 mm (25 µg/disc); MIC = 63–1000 µg/mL; MBC = 195–12,500 µg/mL	[68]
*Alchemilla fissa* Günther and Schummel	*Rosaceae*	Aerial parts	MIC = 4–32 μg/mL	[71]
*Alchemilla glabra* Neygenf.	*Rosaceae*	Aerial parts	MIC = 4–32 μg/mL	[71]
*Alchemilla monticola* Opiz	*Rosaceae*	Aerial parts	MIC = 4–32 μg/mL	[71]
*Alchemilla viridiflora* Rothm.	*Rosaceae*	Aerial parts	MIC = 4–16 μg/mL	[71]
*Alchornea triplinervia* (Spreng.) Müll.Arg.	*Euphorbiaceae*	Aerial parts	MIC = 250 µg/mL	[72]
*Alepidea amatymbica* Eckl. and Zeyh	*Apiaceae*	Roots/rhizomes	IZD = 6.1 ± 6.4 mm	[50,51]
*Alpinia galanga* (L.) Willd. (cited as *Languas galanga*)	*Zingiberaceae*	Tuber	IZD = 21.5 ± 1.9 mm (240 µg/disc)	[59]
*Amphipterygium adstringens* (Schltdl.) Standl.	*Anacardiaceae*	Aerial parts	MIC = 250 µg/mL	[52]
*Anoda cristata* (L.) Schltdl.	*Malvaceae*	Leaves/stem	MIC = 500 µg/mL	[52]
*Artemisia ludoviciana* Nutt. subsp. *mexicana* (Willd. ex Spreng.) Fernald	*Compositae*	Leaves/stem	MIC = 250 µg/mL	[52]
*Aulotandria kamerunensis* (Loes)	*Zingiberaceae*	Rhizome	IZD = 16–22 mm (25 µg/disc)	[68]
*Bidens pilosa* L.	*Compositae*	Leaves	MIC = 128–512 μg/mL	[73]
*Bryophyllum pinnatum* (Lam.) Kurz	*Crassulaceae*	Leaves	MIC = 32 μg/mL; MBC = 256 μg/mL	[74]
*Calandrinia ciliata* (Ruiz and Pav.) DC. (cited as *Calandrinia micrantha*)	*Portulacaceae*	Leaves/Stem	MIC = 250 µg/mL	[52]
*Calophyllum brasiliense* Cambess.	*Clusiaceae*	Bark	MIC = 31 µg/mL; IZD = 7–8 mm (62.5–1000 µg/disc)	[75]
*Calotropis gigantea* (L.) W.T. Aiton	*Apocynaceae*	Leaves	IZD = 9.8 ±1.2 mm (240 µg/disc)	[59]
*Calotropis procera* W.T. Aiton	*Apocynaceae*	Flowers	MIC = 64–256 μg/mL	[49]
*Capsella bursa-pastoris* (L.) Medik.	*Brassicaceae*	Aerial parts	MIC = 62.5 µg/mL	[52]
*Carum carvi* L.	*Apiaceae*	Seeds	MIC = 100 µg/mL	[69]
*Casuarina equisetifolia* L.	*Casuarinaceae*	Fruit	MIC = 128–512 μg/mL	[49]
*Centella asiatica* (L.) Urb.	*Apiaceae*	Whole plant	IZD = 13.0 ± 0.9 mm (240 µg/disc)	[59]
*Chenopodium incisum* Poir. (cited as *Teloxys graveolens*)	*Amaranthaceae*	Aerial parts	MIC = 62.5 µg/mL	[52]
*Chromolaena odorata* (L.) R.M. King and H. Rob.	*Asteraceae*	Leaves	IZD = 25.3 ± 1.6 mm (240 µg/disc)	[59]
*Cistus laurifolius* L.	*Cistaceae*	Flowers	MIC = 62.5–125 µg/mL	[54]
*Colubrina asiatica* (L.) Brongn.	*Rhamnaceae*	Leaves	IZD = 16.3 ± 2.1 mm (240 µg/disc)	[59]
*Combretum molle* R. Br. Ex G. Don	*Combretaceae*	Bark	IZD = 13.1 ± 5.3 mm	[50,51]
*Cosmos caudatus* Kunth	*Asteraceae*	Leaves	IZD = 23.0 ± 0.9 mm (240 µg/disc)	[59]
*Cuminum cyminum* L.	*Apiaceae*	Seed	MIC = 100 µg/mL	[69]
*Curcuma longa* L.	*Zingiberaceae*	*Rhizome*	MIC = 12.5–100 µg/mL	[69]
*Curcuma longa* L./*Zingiber officinale* L.	*Zingiberaceae*	*Rhizome*	MIC = 3.125–100 µg/mL	[69]
*Cymbopogon citratus* (DC.) Stapf	*Poaceae*	Aerial parts	MIC = 31.2 µg/mL	[52]
		Stem	IZD = 28.5 ± 1.5 mm (240 µg/disc)	[59]
*Cyrtocarpa procera* Kunth	*Anacardiaceae*	Bark	MIC = 62.5 µg/mL	[58]
*Derris trifoliata* Lour.	*Leguminosae*	Stem	IZD = 47.0 ± 0.9 mm (240 µg/disc)	[59]
			IZD = 8.5 ± 1.0 mm (240 µg/disc)	[59]
*Dysphania ambrosioides* (L.) Mosyakin and Clemants (cited as *Teloxys ambrosioides*)	*Amaranthaceae*	Aerial parts	MIC = 250–500 µg/mL	[52]
*Elettaria cardamomum* (L.) Maton.	*Zingiberaceae*	Seed	MIC = 3.125-100 µg/mL	[69]
*Emilia coccinea* (Sims) G. Don	*Compositae*	Whole plant	IZD = 6–22 mm (25 µg/disc)	[68]
*Eryngium carlinae* F. Delaroche	*Apiaceae*	Aerial parts	MIC = 31.2 µg/mL	[52]
*Eryngium foetidium* L.	*Apiaceae*	Whole plant	IZD = 6–18 mm (25 µg/disc)	[68]
		Leaves	MIC = 64–512 μg/mL	[73]
*Eucalyptus torelliana* F. Muell.	*Myrtaceae*	Stem bark	MIC = 12.5–200 µg/mL	[76]
*Eupatorium petiolare* Moc. ex DC.	*Compositae*	Aerial parts	MIC = 125 µg/mL	[52]
*Euphorbia hirta* L.	*Euphorbiaceae*	Whole plant	IZD = 6–18 mm (25 µg/disc)	[68]
*Euphorbia umbellata* (Pax) Bruyns	*Euphorbiaceae*	Bark	44.6% inhibition (256 μg/mL)	[77]
*Fagoniaar abica* L.	*Zygophyllaceae*	Whole plant	MIC = 32–256 μg/mL	[49]
*Ficus deltoidea* Jack	*Moraceae*	Leaves	IZD = 12.0 ± 0.6 mm (240 µg/disc)	[59]
*Foeniculum vulgare* Mill. var. *dulce* DC	*Apiaceae*	Seeds	MIC = 50–100 µg/mL	[69]
*Garcinia kola* Heckel	*Guttiferae*	Seeds	IZD = 7.1 ± 5.8 mm	[50,51]
*Galinsoga ciliata* (Raf.) S. F. Blake	*Compositae*	Leaves	MIC = 128–512 μg/mL	[73]
*Gentiana lutea* L.	*Gentianaceae*	Root	MIC = 3.125–100 µg/mL	[69]
*Ginkgo biloba* L.	*Ginkgoaceae*	Leaves	MIC = 100 µg/mL	[69]
*Glycyrrhiza glabra* L.	*Leguminosae*	Root	IDZ = 19 mm (10 mg/mL)	[78]
*Gnaphalium canescens* DC.	*Compositae*	Aerial parts	MIC = 62.5 µg/mL	[52]
*Grindelia inuloides* Willd.	*Compositae*	Aerial parts	MIC = 62.5 µg/mL	[52]
*Haplopappus spinulosus* (Pursh) DC.	*Compositae*		MIC = 125 µg/mL	[52]
*Hesperozygis marifolia* Epling	*Lamiaceae*	Aerial parts	MIC = 62.5 µg/mL	[52]
*Heterotheca inuloides* Cass.	*Compositae*	Aerial parts	MIC = 31.25 µg/mL	[52]
*Hibiscus rosa-sinensis* L.	*Malvaceae*	Stem	IZD = 13.7 ± 1.2 mm (240 µg/disc)	[59]
		Leaves	IZD = 14.3 ± 1.0 mm (240 µg/disc)	[59]
*Hippocratea celastroides* HBK	*Hippocrateace*	Root bark	MIC = 31.25–125 μg/mL	[79]
		Leaves	MIC = 7.81–31.25 μg/mL	[79]
		Stem	MIC = 7.81–15.63 μg/mL	[79]
*Hydrastis canadensis* L.	*Ranunculaceae*	Rhizome	MIC = 0.78–50 µg/mL	[80]
*Illicium verum* Hook. f.	*Schisandraceae*	Fruit	MIC = 50–100 µg/mL	[69]
*Jatropha podagrica* Hook.	*Euphorbiaceae*	Leaves	IZD = 8.0 ± 0.7 mm (240 µg/disc)	[59]
		Stem	IZD = 9.2 ± 0.8 mm (240 µg/disc)	[59]
		Root	IZD = 34.0 ± 2.5 mm (240 µg/disc)	[59]
*Juniperus communis* L.	*Cupressaceae*	Berry	MIC = 25–100 µg/mL	[69]
*Kaempferia galanga* L.	*Zingiberaceae*	Leaves	IZD = 46.0±0.1 mm (240 µg/disc)	[59]
		Tuber	IZD = 11.0 ± 0.6 mm (240 µg/disc)	[59]
*Larrea tridentata* (Sessé and Moc. ex DC.) Coville	*Zygophyllaceae*	Aerial parts	MIC = 62.5 µg/mL	[52]
*Laurus nobilis* L.	*Lauraceae*	Leaves	MIC = 50–100 µg/mL	[69]
*Lavandula angustifolia* Mill.	*Lamiaceae*	Flower	MIC = 100–1000 µg/mL	[69]
*Limnocharis flava* (L.) Buchenau	*Alismataceae*	Leaves	IZD = 11.0 ±1.1 mm (240 µg/disc)	[59]
*Lippia graveolens* Kunth (cited as *Lippia berlandieri*)	*Verbenaceae*	Aerial parts	MIC = 31.2 µg/mL	[52]
*Lithraea molleoides* (Vell.) Engl.	*Anacardiaceae*	Aerial parts	MIC = 18–125 μg/mL	[81]
*Ludwigia repens* J. R. Forst.	*Onagraceae*	Aerial parts	MIC = 500 µg/mL	[52]
*Lycopodium cernua* (L.) Pic. Serm	*Lycopodiaceae*	Whole plant	IZD = 16–22 mm (25 µg/disc; MIC = 63–250 µg/mL; MBC = 195–12500 µg/mL	[68]
*Machaeranthera parviflora* A. Gray	*Compositae*	Aerial parts	MIC = 31.2 µg/mL	[52]
*Machaeranthera riparia* (Kunth) A.G. Jones	*Compositae*	Aerial parts	MIC = 62.5 µg/mL	[52]
*Machaeranthera tanacetifolia* (Kunth) Nees	*Compositae*	Aerial parts	MIC = 125 µg/mL	[52]
*Marantodes pumilum* (Blume) Kuntze (cited as *Labisia pumila*)	*Primulaceae*	Root	IZD = 8.0 ±0.5 mm (240 µg/disc)	[59]
*Marrubium vulgare* L.	*Lamiaceae*	Leaves/stem	MIC = 31.2 µg/mL	[52]
*Melastoma malabathricum* L. *(blue variety*)	*Melastomataceae*	Leaves	IZD = 25.7 ± 0.8 mm (240 µg/disc)	[59]
		Stem	IZD = 18.0 ±0.6 mm (240 µg/disc)	[59]
*Melissa officinalis* L.	*Lamiaceae*	Leaves	MIC = 100 ≥ 100 µg/mL	[69]
*Mentha × piperita* L.	*Lamiaceae*	Leaves	MIC = 25–100 µg/mL	[69]
		Leaves/Stem	MIC = 500 µg/mL	[52]
*Mimosa pudica* L.	*Leguminosae*	Whole plant	IZD = 14.2 ± 1.9 mm (240 µg/disc)	[59]
*Mitrasacme indica* Wight (cited as *Mitrasacme alsinoides*)	*Loganiaceae*	Leaves	IZD = 13.3 ± 2.3 mm (240 µg/disc)	[59]
*Monarda citriodora var. austromontana* (Epling) B. L. Turner. (cited as *Monarda austromontana*)	*Lamiaceae*	Aerial parts	MIC = 125 µg/mL	[52]
*Moussonia deppeana* (Schltdl. and Cham.) Klotzsch ex Hanst.	*Gesneriaceae*	Leaves/stem	MIC = 15.6 µg/mL	[52]
*Myristica fragrans* Houtt.	*Myristicaceae*	Seed	MIC = 3.125–25 µg/mL	[69]
*Neptunia oleracea* Lour.	*Leguminosae*	Leaves	IZD = 28.3 ± 4.1 mm (240 µg/disc)	[59]
*Ocimum basilicum* L.	*Lamiaceae*	Aerial parts	MIC = 31.2 µg/mL	[52]
*Origanum majorana* L.	*Lamiaceae*	Aerial parts	MIC = 50–100 µg/mL	[69]
*Origanum vulgare* L.	*Lamiaceae*	Leaves	MIC = 100 ≥ 100 µg/mL	[69]
*Orthosiphon aristatus* (Blume) Miq. (cited as *Orthosiphon stamineus*)	*Lamiaceae*	Leaves	IZD = 22.0 ± 2.4 mm (240 µg/disc)	[59]
		Stem	IZD = 16.0 ± 0.9 mm (240 µg/disc)	[59]
*Paeonia × suffruticosa* Andrews	*Paeoniaceae*	Root Cortex	IZD = 17 ± 0.08 mm (1 mg/disc)	[82]
*Parkia speciosa* Hassk.	*Leguminosae*	Seed	IZD = 18.0 ± 0.1 mm (240 µg/disc)	[59]
*Passiflora edulis* Sims (cited as *Passiflora incarnata*)	*Passifloraceae*	*Aerial parts*	MIC = 50–100 µg/mL	[69]
*Persicaria minor* (Huds.) Opiz (cited as *Polygonum minus*)	*Polygonaceae*	Leaves	IZD = 15.5 ± 1.1 mm (240 µg/disc)	[59]
*Petroselinum crispum* (Mill.) Fuss	*Apiaceae*	Aerial parts	MIC = 100 ≥ 100 µg/mL	[69]
*Phaeomeria imperialis* (Roscoe) Lindl.	*Zingiberaceae*	Flowers	IZD = 16.3 ± 1.4 mm (240 µg/disc)	[59]
*Phyllanthus niruri* L.	*Phyllanthaceae*	Whole plant	IZD = 29.7 ± 1.4 mm (240 µg/disc)	[59]
*Piper betle* L.	*Piperaceae*	Leaves	IZD = 23.5 ± 0.8 mm (240 µg/disc)	[59]
*Plantago major* L.	*Plantaginaceae*	Aerial parts	MIC = 250 µg/mL	[52]
*Plectranthus amboinicus* (Lour.) Spreng.	*Lamiaceae*	Aerial parts	MIC = 31.2 µg/mL	[52]
*Pluchea indica* (L.) Less.	*Compositae*	Leaves	IZD = 23.0 ± 1.3 mm (240 µg/disc)	[59]
*Poliomintha longiflora* A. Gray	*Lamiaceae*	Leaves/stem	MIC = 250 µg/mL	[52]
*Priva grandiflora* (Ortega) Moldenke	*Verbenaceae*	Aerial parts	MIC = 500 µg/mL	[52]
*Psidium guajava* L.	*Myrtaceae*	Leaves	IZD = 33.0 ± 2.3 mm (240 µg/disc)	[59]
*Quercus rugosa* Née	*Fagaceae*	Leaves	MIC = 125 µg/mL	[52]
*Rosmarinus officinalis* L.	*Lamiaceae*	Leaves	MIC = 12.5–100 µg/mL	[69]
*Ruta chalepensis* L.	*Rutaceae*	Leaves	MIC = 62.5 µg/mL	[52]
*Salvia officinalis* L.	*Lamiaceae*	Leaves	MIC = 25–100 µg/mL	[69]
*Sanguinaria canadensis* L.	*Papaveraceae*	Rhizome	MIC = 12.5–50 µg/mL	[80]
*Scleria woodii var. ornata* (Cherm.) J. Schultze-Motel (cited as *Scleria striatonux*)	*Cyperaceae*	Root	IZD = 6–30 mm (25 µg/disc); MIC = 63–1000 µg/mL; MBC = 195–12,500 µg/mL	[68]
*Scleria verrucossa* (Wild)	*Cyperaceae*	Root	IZD = 4–20 mm (25 µg/disc)	[68]
*Sclerocarya birrea* A. Rich Hochst	*Anacardiaceae*	Stem bark	IZD = 3.0 ± 4.4 mm	[50,51]
			IZD = 17.3 ± 1.6 mm (240 µg/disc)	[59]
*Solanum torvum* Sw.	*Solanaceae*	Seed	IZD = 12.3 ± 0.8 mm (240 µg/disc)	[59]
*Stachys setifera* C. A. Mey.	*Lamiaceae*	Aerial parts	IZD = 38.3 mm (8 mg/disc)	[83]
*Tagetes lucida* Cav.	*Compositae*	Aerial parts	MIC = 500 µg/mL	[52]
*Tanacetum partshenium* (L.) Sch. Bip.	*Compositae*		MIC = 62.5 µg/mL	[52]
*Tapeinochilos ananassae* (Hassk.) K. Schum.	*Costaceae*	Rhizome	IZD = 6–18 mm (25 µg/disc)	[68]
*Tecoma stans* (L.) Juss. ex Kunth	*Bignoniaceae*	Aerial parts	MIC = 500 µg/mL	[52]
*Tillandsia usneoides* L.	*Bromeliaceae*	Aerial parts	MIC = 125 µg/mL	[52]
*Tinospora sinensis* (Lour.) Merr. (cited as *Tinospora cordifolia*)	*Menispermaceae*	Stem	IZD = 13.7 ± 2.7 mm (240 µg/disc)	[59]
*Tithonia diversifolia* (Hemsl.) A.G.	*Compositae*	Aerial parts	MIC = 62.5 µg/mL	[52]
*Verbena carolina* L.	*Verbenaceae*	Aerial parts	MIC = 500–1000 µg/mL	[52]
*Zingiber officinale* Roscoe	*Zingiberaceae*	Rhizome	MIC = 6.25–50 µg/mL	[69]
			IZD = 19.7 ± 1.5 mm (240 µg/disc)	[59]

MIC, minimal inhibitory concentration; MBC, minimal bactericidal concentration; IZD, inhibition zone diameter.

**Table 5:** Plant acetone extracts with anti-*H. pylori* activity.

Species	Family	Parts	Anti-*H. pylori* Potency	Ref.
*Acacia nilotica (L.) Delile*	*Leguminosae*	Leaves	MIC = 8–128 μg/mL	[49]
		Flowers	MIC = 4–64 μg/mL	[49]
*Adhatoda vasica* Nees	*Acanthaceae*	Whole plant	MIC = 16–512 μg/mL	[49]
*Alepidea Amatymbica* Eckl. and Zeyh	*Apiaceae*	Roots/Rhizomes	IZD = 7.0 ± 6.5 mm	[50,51]
*Bridelia micrantha* (Hochst.) Baill.	*Phyllanthaceae*	Bark	IZD = 16–23 mm	[53]
*Calotropis procera* W.T. Aiton	*Apocynaceae*	Leaves	MIC = 32–256 μg/mL	[49]
		Flowers	MIC = 8–128 μg/mL	[49]
*Casuarina equisetifolia* L.	*Casuarinaceae*	Fruit	MIC = 128.0–1024 μg/mL	[49]
*Cocculus hirsutus* (L.) Diels.	*Menispermaceae*	Leaves	IZD = 22–24 mm (200–1000 μg/mL)	[56]
*Combretum molle* R. Br. Ex G. Don *	*Combretaceae*	Bark	MIC_50_ = 0.08–1.25 mg/mL; IZD = 10.7 ± 4.7 mm;	[50,51]
*Desmostachya bipinnata* (L.) Stapf.	*Gramineae*	Whole plant	MIC = 1.3 mg/mL	[84]
*Fagoniaar abica* L.	*Zygophyllaceae*	Whole plant	MIC = 16–128 μg/mL	[49]
*Garcinia kola* Heckel	*Guttiferae*	Seeds	IZD = 8.8 ± 5.2 mm	[50,51]
*Sclerocarya birrea* A. Rich Hochst *	*Anacardiaceae*	Stem bark	MIC_50_ = 0.06–1.25 mg/mL; IZD = 14.7 ± 2.5 mm	[50,51]

* Exhibited remarkable bactericidal activity against *H. pylori*, killing more than 50% of the strains within 18 h at 4× MIC and led to complete elimnation within 24 h; MIC, minimal inhibitory concentration; MIC_50_, minimal inhibitory concentration required to inhibit 50% of cells growth; IZD, inhibition zone diameter.

**Table 6:** Plant chloroform extracts with anti-*H. pylori* activity.

Species	Family	Parts	Anti-*H. pylori* Potency	Ref.
*Calotropis gigantea* (L.) W.T. Aiton	*Apocynaceae*	Leaves	IZD = 14.0 ± 0. 9 mm (240 µg/disc)	[59]
*Cedrus libani* A. Rich	*Pinaceae*	Cones	MIC = 31.2 µg/mL	[54]
*Centaurea solstitialis* L.	*Asteraceae*	Aerial parts	MIC = 1.95 µg/mL	[54]
*Centella asiatica* (L.) Urb.	*Apiaceae*	Whole plant	IZD = 8.2 ± 0.4 mm (240 µg/disc)	[59]
*Chromolaena odorata* (L.) R.M. King and H. Rob.	*Asteraceae*	Leaves	IZD = 27.5 ± 1.0 mm (240 µg/disc)	[59]
*Cistus laurifolius* L.	*Cistaceae*	Flowers	MIC = 1.95 µg/mL	[54]
*Colubrina asiatica* (L.) Brongn.	*Rhamnaceae*	Leaves	IZD = 10.0 ± 0.9 mm (240 µg/disc)	[59]
*Cosmos caudatus* Kunth	*Asteraceae*	Leaves	IZD = 11.7 ± 0.5 mm (240 µg/disc)	[59]
*Cymbopogon citratus* (DC.) Stapf	*Poaceae*	Stem	IZD = 18.0 ± 1.4 mm (240 µg/disc)	[59]
*Derris trifoliata* Lour.	*Leguminosae*	Stem	IZD = 47.0 ± 1.7 mm (240 µg/disc); MIC_50_ = 2 mg/mL MIC_90_ = 4 mg/L	[59]
			IZD = 38.0 ± 1.0 mm (240 µg/disc)	[59]
*Desmos cochinchinensis* Lour.	*Annonaceae*	Leaves	IZD = 30.0 ± 2.1 mm (240 µg/disc)	[59]
*Desmostachya bipinnata* (L.) Stapf.	*Gramineae*	Whole plant	MIC = 5 mg/mL	[84]
*Eucalyptus camaldulensis* Dehnh	*Myrtaceae*	Stem bark	MIC = 25–100 µg/mL	[76]
		Leaves	MIC = 50 µg/mL	[76]
*Eucalyptus torelliana* F. Muell.	*Myrtaceae*	Leaves	MIC = 25–400 µg/mL	[76]
		Stem bark	MIC = 50–100 µg/mL	[76]
*Ficus deltoidea* Jack	*Moraceae*	Leaves	IZD = 10.0 ± 0.6 mm (240 µg/disc)	[59]
*Heterotheca inuloides* Cass.	*Compositae*	Leaves	IZD = 11.2 ± 1.2 mm (240 µg/disc)	[59]
		Stem	IZD = 9.6 ± 0.6 mm (240 µg/disc)	[59]
*Hypericum perforatum* L.	*Hypericaceae*	Aerial parts	MIC = 7.8–31.2 µg/mL	[54]
*Jatropha podagrica* Hook.	*Euphorbiaceae*	Leaves	IZD = 10.0 ± 0.5 mm (240 µg/disc)	[59]
		Root	IZD = 42.0 ± 0.5 mm (240 µg/disc)	[59]
*Kaempferia galanga* L.	*Zingiberaceae*	Leaves	IZD = 66.0 ± 0.1 mm (240 µg/disc)	[59]
		Tuber	IZD = 18.3 ± 1.0 mm (240 µg/disc)	[59]
*Alpinia galanga* (L.) Willd. (cited as *Languas galanga*)	*Zingiberaceae*	Tuber	IZD = 24.2 ± 1.6 mm (240 µg/disc)	[59]
*Limnocharis flava* (L.) Buchenau	*Alismataceae*	Leaves	IZD = 14.0 ± 0.6 mm (240 µg/disc)	[59]
*Melastoma malabathricum* L. (blue variety)	*Melastomataceae*	Leaves	IZD = 22.2 ± 1.3 mm (240 µg/disc)	[59]
		Stem	IZD = 7.2 ± 0.4 mm (240 µg/disc)	[59]
*Mimosa pudica* L.	*Leguminosae*	Whole plant	IZD = 8.8 ± 1.6 mm (240 µg/disc)	[59]
*Mitrasacme indica* Wight (cited as *Mitrasacme alsinoides*)	*Loganiaceae*	Leaves	IZD = 9.5 ± 1.1 mm (240 µg/disc)	[59]
*Momordica charantia* L.	*Cucurbitaceae*	Fruits	MIC = 31.2–125 µg/mL	[54]
*Neptunia oleracea* Lour.	*Leguminosae*	Leaves	IZD = 10.7 ± 2.0 mm (240 µg/disc)	[59]
*Orthosiphon aristatus* (Blume) Miq. (cited as *Orthosiphon stamineus*)	*Lamiaceae*	Leaves	IZD = 18.3 ± 2.2 mm (240 µg/disc)	[59]
		Stem	IZD = 11.3 ± 1.0 mm (240 µg/disc)	[59]
*Paeonia × suffruticosa* Andrews	*Paeoniaceae*	Root Cortex	IZD = 23.9–26.7 mm (1–10 mg/disc)	[82]
*Parkia speciosa* Hassk.	*Leguminosae*	Seed	IZD = 26.0 ± 0.6 mm (240 µg/disc)	[59]
*Phaeomeria imperialis* (Roscoe) Lindl.	*Zingiberaceae*	Flowers	IZD = 14.0 ± 0.6 mm (240 µg/disc)	[59]
*Phyllanthus niruri* L.	*Phyllanthaceae*	Whole plant	IZD = 9.8 ± 0.8 mm (240 µg/disc)	[59]
*Piper betle* L.	*Piperaceae*	Leaves	IZD = 25.8 ± 0.8 mm (240 µg/disc)	[59]
*Pluchea indica* (L.) Less.	*Compositae*	Leaves	IZD = 11.0 ± 0.6 mm (240 µg/disc)	[59]
*Persicaria minor* (Huds.) Opiz (cited as *Polygonum minus*)	*Polygonaceae*	Leaves	IZD = 12.3 ± 0.8 mm (240 µg/disc)	[59]
*Psidium guajava* L.	*Myrtaceae*	Leaves	IZD = 10.0 ± 0.6 mm (240 µg/disc)	[59]
*Sambucus ebulus*	*Adoxaceae*	Aerial parts	MIC = 31.2 µg/mL	[54]
*Sesbania grandiflora* (L.) Pers.	*Leguminosae*	Leaves	IZD = 8.8 ± 1.1 mm (240 µg/disc)	[59]
*Solanum torvum* Sw.	*Solanaceae*	Seed	IZD = 8.7 ± 0.0 mm (240 µg/disc)	[59]
*Tinospora sinensis* (Lour.) Merr. (cited as *Tinospora cordifolia*)	*Menispermaceae*	Stem	IZD = 19.2 ± 5 mm (240 µg/disc)	[59]
*Zingiber officinale* Roscoe	*Zingiberaceae*	Rhizome	IZD = 41.5 ± 7.0 mm (240 µg/disc)	[59]

MIC, minimal inhibitory concentration; MIC_50_ and MIC_90_, minimal inhibitory concentration required to inhibit 50% and 90% of cells growth, respectively; IZD, inhibition zone diameter.

**Table 7:** Plant petroleum ether extracts with anti-*H. pylori* activity.

Species	Family	Parts	Anti-*H. pylori* Potency	Ref.
*Calotropis gigantea* (L.) W.T. Aiton	*Apocynaceae*	Leaves	IZD = 13.2 ± 0.8 mm (240 µg/disc)	[59]
*Centella asiatica* (L.) Urb.	*Apiaceae*	Whole plant	IZD = 8.5 ± 0.6 mm (240 µg/disc)	[59]
*Chromolaena odorata* (L.) R.M. King and H. Rob.	*Asteraceae*	Leaves	IZD = 20.3 ± 1.4 mm (240 µg/disc)	[59]
*Colubrina asiatica* (L.) Brongn.	*Rhamnaceae*	Leaves	IZD = 11.0 ± 0.9 mm (240 µg/disc)	[59]
*Cosmos caudatus* Kunth	*Asteraceae*	Leaves	IZD = 16.0 ± 0.6 mm (240 µg/disc)	[59]
*Cymbopogon citratus* (DC.) Stapf	*Poaceae*	Stem	IZD = 29.5 ± 1.5 mm (240 µg/disc)	[59]
*Derris trifoliata* Lour.	*Leguminosae*	Stem	IZD = 42.0 ± 0.9 mm (240 µg/disc); MIC_50_ = 1 mg/mL; MIC_90_ = 2 mg/L	[59]
			IZD = 42.0 ± 1.0 mm (240 µg/disc)	[59]
*Desmostachya bipinnata* (L.) Stapf.	*Gramineae*	Whole plant	MIC = 1.5 mg/mL	[84]
*Ficus deltoidea* Jack	*Moraceae*	Leaves	IZD = 8.0 ± 0.1 mm (240 µg/disc)	[59]
*Heterotheca inuloides* Cass.	*Compositae*	Leaves	IZD = 11.5 ± 1.1 mm (240 µg/disc)	[59]
		Stem	IZD = 13.2 ± 0.1 mm (240 µg/disc)	[59]
*Jatropha podagrica* Hook.	*Euphorbiaceae*	Leaves	IZD = 13.0 ± 1.1 mm (240 µg/disc)	[59]
		Stem	IZD = 15.5 ± 1.4 mm (240 µg/disc)	[59]
		Root	IZD = 47.3 ± 3.1 mm (240 µg/disc)	[59]
*Kaempferia galanga* L.	*Zingiberaceae*	Leaves	IZD = 62.0 ± 0.1 mm (240 µg/disc)	[59]
		Tuber	IZD = 18.3 ± 1.0 mm (240 µg/disc)	[59]
*Alpinia galanga* (L.) Willd. (cited as *Languas galanga*)	*Zingiberaceae*	Tuber	IZD = 39.3 ± 2.1 mm (240 µg/disc)	[59]
*Limnocharis flava* (L.) Buchenau	*Alismataceae*	Leaves	IZD = 24.0 ± 0.6 mm (240 µg/disc)	[59]
*Melastoma malabathricum* L. (blue variety)	*Melastomataceae*	Leaves	IZD = 14.0 ± 2.3 mm (240 µg/disc)	[59]
		Stem	IZD = 10.5 ± 0.8 mm (240 µg/disc)	[59]
*Mimosa pudica* L.	*Leguminosae*	Whole plant	IZD = 8.5 ± 0.6 mm (240 µg/disc)	[59]
*Mitrasacme indica* Wight (cited as *Mitrasacme alsinoides* R. Br.)	*Loganiaceae*	Leaves	IZD = 11.0 ± 0.6 mm (240 µg/disc)	[59]
*Neptunia oleracea* Lour.	*Leguminosae*	Leaves	IZD = 10.5 ± 0.8 mm (240 µg/disc)	[59]
*Orthosiphon aristatus* (Blume) Miq. (cited as *Orthosiphon stamineus*)	*Lamiaceae*	Leaves	IZD = 17.7 ± 2.8 mm (240 µg/disc)	[59]
		Stems	IZD = 12.7 ± 0.5 mm (240 µg/disc)	[59]
*Parkia speciosa* Hassk.	*Leguminosae*	Seeds	IZD = 10.5 ± 0.8 mm (240 µg/disc)	[59]
*Pereskia sacharosa* Griseb.	*Cactaceae*	Leaves	IZD = 13.3 ± 0.5 mm (240 µg/disc)	[59]
*Etlingera elatior* (Jack) R.M.Sm. (cited as *Phaeomeria imperialis*)	*Zingiberaceae*	Flowers	IZD = 18.0 ± 1.1 mm (240 µg/disc)	[59]
*Phyllanthus niruri* L.	*Phyllanthaceae*	Whole plant	IZD = 14.0 ± 1.6 mm (240 µg/disc)	[59]
*Piper betle* L.	*Piperaceae*	Leaves	IZD = 54.2 ± 0.8 mm (240 µg/disc)	[59]
*Pluchea indica* (L.) Less.	*Compositae*	Leaves	IZD = 13.7 ± 1.9 mm (240 µg/disc)	[59]
*Persicaria minor* (Huds.) Opiz (cited as *Polygonum minus*)	*Polygonaceae*	Leaves	IZD = 15.5 ± 0.6 mm (240 µg/disc)	[59]
*Psidium guajava* L.	*Myrtaceae*	Leaves	IZD = 8.5 ± 0.8 mm (240 µg/disc)	[59]
*Sesbania grandiflora* (L.) Pers.	*Leguminosae*	Leaves	IZD = 10.8 ± 1.0 mm (240 µg/disc)	[59]
*Solanum torvum* Sw.	*Solanaceae*	Seeds	IZD = 11.0 ± 0.9 mm (240 µg/disc)	[59]
*Tinospora sinensis* (Lour.) Merr. (cited as *Tinospora cordifolia*)	*Menispermaceae*	Stems	IZD = 10.7 ± 0.8 mm (240 µg/disc)	[59]
*Zingiber officinale* Roscoe	*Zingiberaceae*	Rhizome	IZD = 33.3 ± 1.6 mm (240 µg/disc)	[59]

MIC, minimal inhibitory concentration; MIC_50_, minimal inhibitory concentration required to inhibit 50% of cells growth; IZD, inhibition zone diameter.

**Table 8:** Plant methanol/water, ethanol/water, methanol/petroleum, and methanol/dichloromethane extracts with anti-*H. pylori* activity.

Species	Family	Parts	Anti-*H. pylori* Potency	Ref.
**Methanol/Water (70:30, *v*/*v*)**				
*Acacia seyal* Delile	*Leguminoseae*	Stem	MIC = 20 mg/mL	[84]
		Leaves	MIC = 20 mg/mL	[84]
*Alhagi maurorum* Medik.	*Leguminoseae*	Whole plant	MIC = 0.79 mg/mL	[84]
*Bidens bipinnata* L.	*Compositae*	Whole plant	MIC = 25 mg/mL	[84]
*Capparis spinose* L.	*Capparaceae*	Aerial parts	MIC = 10 mg/mL	[84]
*Casimiroa edulis* Llave and Lex	*Rutaceae*	Unripe fruit	MIC = 20 mg/mL	[84]
*Centaurea alexandrina* Delile	*Compositae*	Whole plant	MIC = 80 mg/mL	[84]
*Centaurea pelia* DC.	*Compositae*	ND	MIC = 0.625–5 mg/mL	[85]
*Centaurea thessala* Hausskn. ssp. *drakiensis* (Freyn and Sint.) Georg	*Compositae*	ND	MIC = 0.625–5 mg/mL	[85]
*Cerastium candidisimum* L.	*Caryophyllaceae*	ND	MIC = 0.625–2.5 mg/mL	[85]
*Chamomilla recutita* (L.) Rauschert	*Compositae*	ND	MIC = 0.625–2.5 mg/mL	[85]
*Cleome africana* Botsch.	*Cleomaceae*	Whole plant	MIC = 0.158 mg/mL	[84]
*Conyza albida* Willd. ex Spreng.	*Asteraceae*	ND	MIC = 0.625–2.5 mg/mL	[85]
*Conyza bonariensis* (L.) Cronquist.	*Asteraceae*	ND	MIC = 0.625–2.5 mg/mL	[85]
*Cota palaestina* Reut. ex Unger and Kotschy (cited as *Anthemis melanolepis*)	*Compositae*	ND	MIC = 0.625–2.5 mg/mL	[85]
*Desmostachya bipinnata* (L.) Stapf.	*Gramineae*	Whole plant	MIC = 0.040 mg/mL	[84]
*Diplotaxis acris* (Forssk.) Boiss.	*Cruciferae*	Whole plant	MIC = 10 mg/mL	[84]
*Dittrichia viscosa* (L.) Greuter subsp. *revoluta*	*Asteraceae*	ND	MIC = 0.625–2.5 mg/mL	[85]
*Euphorbia retusa* Forssk.	*Euphorbiaceae*		MIC = 2.5 mg/mL	[84]
*Glossostemon brugueiri* Desf.	*Sterculiaceae*	Root	MIC = 10 mg/mL	[84]
		Leaves	MIC = 25 mg/mL	[84]
*Hamada elegans* (Bunge) Botsch.	*Chenopodiaceae*	Whole plant	MIC = 10 mg/mL	[84]
*Haplophyllum tuberculatum* (Forssk.) A. Juss.	*Rutaceae*	Whole plant	MIC = 1.58 mg/mL	[84]
*Lythrum salicaria* L.*	*Lythraceae*	Aerial parts	IZD = 17 ± 0.08 mm (500 mg/mL)	[86]
*Marrubium vulgare* L.	*Lamiaceae*	Whole plant	MIC = 0.251 mg/mL	[84]
*Ocimum basilicum* L.	*Lamiaceae*	Aerial parts	MIC = 0.625–5 mg/mL	[85]
*Origanum dictamnus* L.	*Lamiaceae*	Aerial parts	MIC = 0.625–5 mg/mL	[85]
*Origanum majorana* L.	*Lamiaceae*	Aerial parts	MIC = 0.625–5 mg/mL	[85]
*Origanum vulgare* L.	*Lamiaceae*	Leaves	MIC = 0.625–2.5 mg/mL	[85]
*Schouwia thebaica* Webb.	*Brassicaceae*	Whole plant	MIC = 25 mg/mL	[84]
*Sisymbrium irio* L.	*Brassicaceae*	Whole plant	MIC = 0.074 mg/mL	[84]
*Stachys alopecuros* (L.) Benth.	*Lamiaceae*	Aerial parts	MIC = 0.625–2.5 mg/mL	[85]
*Thymbra capitata* (L.) Cav. (cited as *Thymus capitatus*)	*Lamiaceae*	Whole plant	MIC = 12.5 mg/mL	[84]
*Trifolium alexandrinum* L.	*Leguminosae*	Whole plant	MIC = 25 mg/mL	[84]
**Ethanol/Water (70:30, *v*/*v*)**				
*Calophyllum brasiliense* Cambess.	*Clusiaceae*	Bark	MIC = 31 µg/mL; IZD = 8–14 mm (62.5–1000 µg/disc)	[75]
*Cocculus hirsutus* (L.) Diels.	*Menispermaceae*	Leaves	IZD = 26 mm (200–1000 μg/mL)	[56]
*Fridericia chica* (Bonpl.) L. G. Lohmann (cited as *Arrabidaea chica*)	*Bignoniaceae*	Fresh leaves	12.5	[87]
*Hancornia speciosa* Gomez	*Apocynaceae*	Bark	MIC = 125 µg/mL	[88]
**Methanol/Petroleum (1:1)**				
*Carum bulbocastanum* (L.) Koch.	*Apiaceae*	Fruit	MIC = 31.25–250 µg/mL	[46]
*Carum carvi* L.	*Apiaceae*	Fruit	MIC = 31.25–125 µg/mL	[46]
*Glycyrrhiza glabra* Linn	*Leguminosae*	Root	MIC = 15.6–250 µg/mL	[46]
*Mentha longifolia* (L). Huds.	*Lamiaceae*	Aerial parts	MIC = 31.25–125 µg/mL	[46]
*Salvia limbata* C. A. Mey.	*Lamiaceae*	Aerial parts	MIC = 125–250 µg/mL	[46]
*Salvia sclarea* L.	*Lamiaceae*	Aerial parts	MIC = 125–500 µg/mL	[46]
*Trachyspermum ammi* (L.) Sprague (cited as *Trachyspermum copticum*)	*Apiaceae*	Aerial parts	MIC = 31.25–250 µg/mL	[46,89]
*Xanthium strumarium* subsp. *brasilicum* (Vell.) O. Bolòs and Vigo (cited as *Xanthium brasilicum*)	*Compositae*	Aerial parts	MIC = 31.25–250 µg/mL	[46,89]
*Ziziphora clinopodioides* Lam.	*Lamiaceae*	Aerial parts	MIC = 31.25–125 µg/mL	[46]
**Methanol/Dichloromethan**				
*Cyrtocarpa procera* Kunth	*Anacardiaceae*	Bark	MIC = 62.5 µg/mL	[58]

* Methanol/water (80:20, *v*/*v*); ND, not defined; MIC, minimal inhibitory concentration; IZD, inhibition zone diameter.

**Table 9:** Plant cyclohexane, dichloromethane, ethyl acetate, n-Butanol, n-Hexane, and other extracts with anti-*H. pylori* activity.

Species	Family	Parts	Anti-*H. pylori* Potency	Ref.
**Cyclohexane**				
*Alchemilla fissa* Günther and Schummel	*Rosaceae*	Aerial parts	MIC = 64–256 μg/mL	[71]
*Alchemilla glabra* Neygenf.	*Rosaceae*	Aerial parts	MIC = 64–256 μg/mL	[71]
*Alchemilla monticola* Opiz	*Rosaceae*	Aerial parts	MIC = 8–64 μg/mL	[71]
*Alchemilla viridiflora* Rothm.	*Rosaceae*	Aerial parts	MIC = 64–256 μg/mL	[71]
**Dichloromethane**				
*Alchemilla fissa* Günther and Schummel	*Rosaceae*	Aerial parts	MIC = 64–256 μg/mL	[71]
*Alchemilla glabra* Neygenf.	*Rosaceae*	Aerial parts	MIC = 64–256 μg/mL	[71]
*Alchemilla monticola* Opiz	*Rosaceae*	Aerial parts	MIC = 16–64 μg/mL	[71]
*Alchemilla viridiflora* Rothm.	*Rosaceae*	Aerial parts	MIC = 16–128 μg/mL	[71]
*Calophyllum brasiliense* Cambess.	*Clusiaceae*	Bark	MIC = 125 µg/mL; IZD = 7–10 mm (62.5–1000 µg/disc)	[75]
*Cyrtocarpa procera* Kunth	*Anacardiaceae*	Bark	MIC = 15.6 µg/mL	[58]
**Ethyl acetate**				
*Alepidea Amatymbica* Eckl. and Zeyh	*Apiaceae*	Roots/rhizomes	IZD = 8.5 ± 4.8 mm	[50,51]
*Bidens pilosa* L.	*Compositae*	Leaves	MIC = 128–512 μg/mL	[73]
*Bridelia micrantha* (Hochst.) Baill.	*Phyllanthaceae*	Bark	IZD = 12–20 mm; MIC_50_ = 4.8–156 µg/mL; MIC_90_ = 4.8–2500 µg/mL	[53]
*Calophyllum brasiliense* Cambess.	*Clusiaceae*	Bark	MIC = 125 µg/mL; IZD = 7–8 mm (62.5–1000 µg/disc)	[75]
*Combretum molle* R. Br. Ex G. Don	*Combretaceae*	Bark	IZD =10.7 ± 4.7 mm	[50,51]
*Desmostachya bipinnata* (L.) Stapf.	*Gramineae*	Whole plant	MIC = 0.79 mg/mL	[84]
*Eryngium foetidium* (Linn)	*Apiaceae*	Leaves	MIC = 128–512 μg/mL	[73]
*Garcinia kola* Heckel	*Guttiferae*	Seeds	IZD = 5.1 ± 4.6 mm	[50,51]
*Galinsoga ciliata* (Raf.) S. F. Blake	*Compositae*	Leaves	MIC = 128–512 μg/mL	[73]
*Geranium wilfordii* Maxim	*Geraniaceae*	Aerial parts	MIC = 30 µg/mL	[90]
*Paeonia × suffruticosa* Andrews	*Paeoniaceae*	Root Cortex	IZD = 14.1–19.9 mm (1–10 mg/disc)	[82]
*Physalis alkekengi* L. *var. franchetii* (Mast.) Makino	*Solanaceae*	Aerial parts	MIC = 500 μg/mL	[91]
*Sclerocarya birrea* A. Rich Hochst	*Anacardiaceae*	Stem bark	IZD = 13.2 ± 2.8 mm	[50,51]
**n-Butanol**				
*Centaurea solstitialis* L. *subsp. solstitialis*	*Asteraceae*	Aerial parts	MIC = 31.2 µg/mL	[54]
*Cistus laurifolius* L.	*Cistaceae*	Flowers	MIC = 62.5–125 µg/mL	[54]
*Hypericum perforatum* L.	*Hypericaceae*	Aerial parts	MIC = 15.6–31.2 µg/mL	[54]
*Momordica charantia* L.	*Cucurbitaceae*	Fruits	MIC = 62.5 µg/mL	[54]
**n-Hexane**				
*Calophylum brasiliense* Cambess.	*Clusiaceae*	Bark	IZD = 7–14 mm (100–400 μg/disc)	[92]
			IZD =7–8 mm (62.5–1000 µg/disc)	[75]
			IZD = 14 mm (400 mg/mL) MIC = 31 µg/mL	[75]
*Cyrtocarpa procera* Kunth	*Anacardiaceae*	Bark	MIC = 7.81 µg/mL	[58]
*Eucalyptus camaldulensis* Dehnh	*Myrtaceae*	Stem bark	MIC = 25–200 µg/mL	[76]
		Leaves	MIC = 50 µg/mL	[76]
*Eucalyptus torelliana* F. Muell.	*Myrtaceae*	Leaves	MIC = 25–50 µg/mL	[76]
		Stem bark	MIC = 25–200 µg/mL	[76]
*Mitrella kentii* (Bl.) Miq	*Annonaceae*	Bark	MIC = 125 μg/mL	[93]
*Paeonia × suffruticosa* Andrews	*Paeoniaceae*	Root Cortex	IZD = 29.9–31.3 mm (1–10 mg/disc)	[82]
**Others**				
*Camellia sinensis* (L.) Kuntze	*Theaceae*	Young shoots	IZD = 22.5 mm (20–60 μg/disc) MBC = 4 mg/mL	[94]
			IZD = 18 mm (20–60 μg/disc) MBC = 5.5 mg/mL	[94]
*Chenopodium ambrosioides* L.	*Amaranthaceae*	Aerial parts	MIC = 16 mg/L *	[95]

* 1 and 2 × MIC completely inhibited *H. pylori* growth at 24 h; MIC, minimal inhibitory concentration; MIC_50_, minimal inhibitory concentration required to inhibit 50% of cells growth; MBC, minimal bactericidal concentration; IZD, inhibition zone diameter.

**Table 10:** Bioactive compounds with anti-*H. pylori* activity.

Plant Species	Bioactive Compounds	Anti-*H. pylori* Potency (MIC)	Ref.
*Allium sativum* L. (cloves)	Allicin (garlic poder)	4 μg/mL	[96]
	Allicin	6 μg/mL	[96]
	Diallyl disulfide	100–200 μg/mL	[96]
	Diallyl tetrasulfide	3–6 μg/mL	[96]
*Convolvulus austro-aegyptiacu* Abdallah and Saad (aerial parts)	Scopoletin	50–200 µg/mL	[67]
	Scopolin	50–100 µg/mL	[67]
*Glycyrrhiza glabra* L. (roots)	Licoricidin	6.25–12.5 µg/mL	[78]
	Licoisoflavone	6.25 µg/mL	[78]
	Fuscaxanthone I	15.2–122.0 μM	[97]
	Beta-Mangostin	18.3–147.3 μM	[97]
	Fuscaxanthone A	16.3–131.2 μM	[97]
	Cowanin	16.3–130.6 μM	[97]
	Cowaxanthone	4.6–152.3 μg/mL	[97]
	Alpha-Mangostin	19.0–76.1 μM	[97]
	Cowanol	15.7–126.4 μM	[97]
	Isojacareubin	23.9 μM	[97]
	Fuscaxanthone G	16.3–130.6 μM	[97]
	Nigrolineabiphenyl B	56.5–226.3 μM	[97]
	1,3,5,6-Tetrahydroxyxanthone	29.9–240.3 μM	[97]
	Vokensiflavone	14.4–115.7 μM	[97]
	Morelloflavone	14.0–112.3 μM	[97]
*Hydrastis canadensis* L. (rhizomes)	Berberine	0.78–25 µg/mL	[80]
	β-Hydrastine	25–100 µg/mL	[80]
*Sanguinaria canadensis* L. (rhizomes)	Sanguinarine	6.25–50 µg/mL	[80]
	Chelerythrine	25–100 µg/mL	[80]
	Protopine	25 ≥ 100 µg/mL	[80]
*Tinospora sagittata* Gagnep. (aerial parts)	Palmatine	3.12–6.25 μg/mL	[42]

MIC, minimal inhibitory concentration.

**Table 11:** Urease inhibitory potential of plant extracts.

Plant Species	Parts	Extraction Solvent	Concentration Tested	Urease Inhibition	Ref.
*Acacia nilotica* (L.) Delile	Leaves	Methanol	8–128 μg/mL	8.21–88.21%	[49]
	Flowers	Acetone	8–128 μg/mL	9.20–86.56%	[49]
*Calotropis procera* (Aiton) W.T. *Aiton*	Leaves	Methanol	16–256 μg/mL	12.23–48.22%	[49]
	Leaves	Acetone	32–256 μg/mL	7.23–58.21%	[49]
	Flowers	Acetone	8–128 μg/mL	9.33–68.21%	[49]
*Camellia sinensis* (L.) Kuntze	Young shoots	Methanol: water (62.5:37.5 *v*/*v*) non-fermented extract	2.5 mg/mL	100% Ure A and B	[94]
		Methanol: water (62.5:37.5 *v*/*v*)) semifermented extract	3.5 mg/mL	100% Ure A and B	[94]
*Casuarina equisetifolia* L.	Fruit	Methanol	128–512 μg/mL	12.21–86.21%	[49]
*Chamomilla recutita* (L.) Rauschert	Flowers	Olive oil	31.25–250 mg/mL	Inhibited urease production	[105]
*Euphorbia umbellata (Pax) Bruyns*	Bark	Methanol	1024 μg/mL	78.6%	[77]
*Peumus boldus* Mol.	Leaves	Water		IC_50_ = 23.4 μg GAE/mL	[61]
*Teminalia chebula Retz*	Fruit	Water	1–2.5 mg/mL	Inhibited urease activity	[63]

IC50, 50% inhibitory concentration. GAE, gallic acid equivalents.
